# A phase III trial comparing CHOP to PMitCEBO with or without G-CSF in patients aged 60 plus with aggressive non-Hodgkin's lymphoma

**DOI:** 10.1038/sj.bjc.6602975

**Published:** 2006-02-28

**Authors:** C Burton, D Linch, P Hoskin, D Milligan, M J S Dyer, B Hancock, P Mouncey, P Smith, W Qian, K MacLennan, A Jack, A Webb, D Cunningham

**Affiliations:** 1 University College London and CRUK Clinical Trials Centre, 222 Euston Road, London NW1 2DA, UK; 2 Mount Vernon Hospital, Rickmansworth Road, Northwood HA6 2RN, UK; 3 Birmingham Heartlands Hospital, Bordesley Green East, Birmingham B9 5SS, UK; 4 University of Leicester, Lancaster Road, Leicester LE1 9HN, UK; 5 Weston Park Hospital, Whitham Road, Sheffield S10 2SJ, UK; 6 MRC Clinical Trials Centre, 222 Euston Road, London NW1 2DA, UK; 7 Leeds General Infirmary, Great George Street, Leeds LS1 3EX, UK; 8 Department of Oncology, Royal Marsden Hospital, Downs Road, Sutton, Surrey SM2 5PT, UK

**Keywords:** elderly, non-Hodgkin's lymphoma, CHOP, PMitCEBO, G-CSF

## Abstract

The management of older patients with aggressive non-Hodgkin's lymphoma presents a challenge to the physician. Age is a poor prognostic indicator, due to reduced ability to tolerate and maintain dose-intensive chemotherapy. Generally, older patients demonstrate a lower response rate, reduced survival and increased toxicity, although the majority of large randomised trials exclude older patients. This randomised trial was conducted in patients 60 years or over to compare CHOP (cyclophosphamide 750 mg m^−2^, doxorubicin 50 mg m^−2^, vincristine 1.4 mg m^−2^, prednisolone 100 mg) with PMitCEBO (mitoxantrone 7 mg m^−2^, cyclophosphamide 300 mg m^−2^, etoposide 150 mg m^−2^, vincristine 1.4 mg m^−2^, bleomycin 10 mg m^−2^ and prednisolone 50 mg). Due to the myelosuppressive nature of these regimens, patients were also randomised to the addition of G-CSF. The formal results of this trial with long-term follow-up are now reported. Data were analysed to assess efficacy and toxicity. Overall response rate was 84% in the CHOP arm and 83% in the PMitCEBO arm, with overall response rates of 83% for the use of G-CSF and 84% for no G-CSF. At median 44 months follow-up, there was no significant difference in failure-free, progression-free or overall survival between the CHOP and PMitCEBO arms. At 3 years, the actuarial failure-free survival was 44% in CHOP recipients and 42% in PMitCEBO recipients and the 3-year actuarial overall survival was 46% and 45% respectively. There was no significant difference in the failure-free, progression-free or overall survival with the addition of G-CSF.

The reported incidence of aggressive non-Hodgkin's lymphomas (NHL) has doubled in recent decades, and this increase has been predominantly observed in older patients (McNally *et al*, 1999; 2001 Census; Muller *et al*, 2005). Advanced age is an independent poor prognostic factor, with inability to tolerate chemotherapy and maintain dose intensity (International NHL Prognostic Factors Project, 1993; NHL Classification Project, 1997; Bastion *et al*, 1997). Higher treatment-related mortality has also been reported in older patients using the CHOP (cyclophosphamide, doxorubicin, vincristine and prednisolone) regimen (Armitage and Potter, 1984). For these reasons, specific regimens have been developed for treating elderly patients, involving dosage reductions and shortening of the period of treatment (Meyer *et al*, 1995; Sonneveld *et al*, 1995). These curtailed regimens have tended to result in a lower toxic death rate but also a lower complete response (CR) rate.

The British National Lymphoma Investigation (BNLI) group have had considerable experience with the 12 weekly PACEBOM regimen (prednisolone, adriamycin, cyclophosphamide, etoposide, bleomycin, vincristine, methotrexate), which alternates myelosuppressive and non-myelosuppressive agents (Sweetenham *et al*, 1991). In a randomised trial in patients under the age of 70 years, PACEBOM was shown to be as efficacious as CHOP (Linch *et al*, 2000). One potential advantage of the PACEBOM regimen is the lower planned total dose of anthracycline (30% reduction), which might be particularly relevant to the older patient. Several recent studies have shown that anthracycline-related cardiotoxicity is more frequent in older patients (Limat *et al*, 2003; Swain *et al*, 2003). Swain *et al* suggested that the threshold for cardiac damage is doxorubicin 400 mg m^−2^. Limat *et al* suggested it is even lower. The tolerability of PACEBOM in the older population though was frequently limited by mucositis, and subsequently the methotrexate was omitted. This PACEBO regimen was then compared in a randomised trial, in elderly patients with histologically aggressive NHL, with PMitCEBO, in which the 35 mg m^−2^ adriamycin was replaced by 7 mg m^−2^ mitoxantrone, an anthracenedione derivative (Mainwaring *et al*, 2001). This trial suggested that PMitCEBO was superior to PACEBO, with an encouraging 40% survival at 4 years. This contrasts with the findings in another trial where the substitution of adriamycin by mitoxantrone had a negative impact (Sonneveld *et al*, 1995). In the trial reported here, PMitCEBO has been compared with standard CHOP.

Both the PMitCEBO and CHOP regimens result in considerable myelosuppression in the elderly population and the value of using granulocyte colony-stimulating factor (G-CSF) to mitigate against the chemotherapy induced neutropenia was also explored. Previous studies in lymphoma have suggested that the use of G-CSF results in less granulocyte suppression, less infection and hospitalisation, possibly allowing a higher dose intensity with improved response rates and with significant economic benefits (Pettengell *et al*, 1992; Gerhartz *et al*, 1993; Bertini *et al*, 1994; Zagonel *et al*, 1994; Niitsu and Umeda, 1995; Silvestri *et al*, 1995). Few previous studies have been adequately powered, however, to evaluate the impact of G-CSF use on survival. In the trial reported here, the use of G-CSF in combination with chemotherapy has been compared with chemotherapy alone.

The mature results of this study of 784 patients are presented in this paper.

## PATIENTS AND METHODS

### Patient selection

Patients were eligible if aged 60 years or over with previously untreated, bulky stage IA or stages IB-IV aggressive NHL. There was no upper age limit. Between 1997 and 1999 patients with a histological diagnosis of diffuse mixed cell, diffuse large cell or diffuse immunoblastic lymphoma according to the Working Formulation were included. From 2000 the histological diagnosis was made according to the World Health Organisation and patients with diffuse large B-cell lymphoma were included. Central review including immunophenotyping was performed at a later date in 627 cases and in 52 (8%) the patient was considered ineligible on histological criteria either because of a change in diagnosis or because of inadequate material. The analyses performed were on an intention to treat basis and patients were not excluded after randomisation.

Full clinical staging with routine haematological and biochemical blood tests, computed tomography (CT) imaging of the chest, abdomen and pelvis and bone marrow biopsy were performed. Any patient with central nervous system disease, lymphoblastic or Burkitt lymphoma was excluded from the trial. Patients with significant renal, hepatic or cardiac dysfunction were excluded (creatinine>150 *μ*mol l^−1^, bilirubin>35 *μ*mol l^−1^, left ventricular ejection fraction<50%, respectively). No patients with medical conditions, other than aggressive non-Hodgkin's lymphoma, prohibiting intensive therapy were included and no patient had received systemic treatment for cancer in the preceding 5 years.

### Trial design

Between October 1997 and September 2003, 784 newly diagnosed patients from 92 centres in UK were entered into the trial. The trial had a 2 × 2 factorial design; randomisations between CHOP and PMitCEBO (comparing all patients randomised to CHOP with all patients randomised to PMitCEBO) and the addition or not of G-CSF (comparing all patients randomised to G-CSF with all those patients not randomised to G-CSF).

The CHOP regimen was given 3-weekly for a maximum of eight cycles and the PMitCEBO regimen weekly for a maximum of 16 weeks (Table 1). Prophylactic G-CSF (Lenograstim) 263 *μ*g day^−1^ was administered if randomised to G-CSF from days 8 to 14 in the CHOP arm and days 6 to 12 in the PMitCEBO arm. All patients received allopurinol for weeks 1–3 and co-trimoxazole prophylaxis week 1 to treatment end plus 2 weeks. Patients treated with CHOP chemotherapy were assessed clinically and by CT imaging after 2, 4 and 6 cycles. If CR or non-progressive partial remission (PR) was achieved, patients were treated with six cycles of CHOP. If in a progressive PR at six cycles, two further cycles of CHOP were given. For patients treated with PMitCEBO, initial response was assessed clinically and by CT imaging after 4, 8 and, if applicable, 12 weeks. Patients were treated to CR or nonprogressive PR, plus a further 4 weeks of chemotherapy. Methotrexate 12.5 mg intrathecally was given as six doses at weekly intervals to patients with peripheral blood, orbital, testicular or facial sinus disease.

Dose reduction was identical for both chemotherapy regimens and based on Southwest Oncology Group studies (Fisher *et al*, 1993). No dose modifications were made on the first cycle based on blood counts. Dose modifications for haematological toxicity for subsequent cycles were made as presented in Table 2. No dosage reduction was made for low haemoglobin. Blood transfusions were given to maintain haemoglobin >10 g dl^−1^. The dose of anthracycline was also reduced by 50 or 75% if the serum bilirubin was raised to 35–50 or >50 *μ*mol l^−1^, respectively. Bleomycin was discontinued if there were any clinical signs or radiological evidence of pulmonary infiltration/fibrosis developing. Bleomycin was also discontinued if severe skin toxicity developed. Vincristine was reduced by 50% in patients with grade 2 motor toxicity (mild, objective weakness, but no significant impairment of function) and grade 3 sensory toxicity (severe objective sensory loss or paraesthesiae interfering with function). Vincristine was completely omitted for higher grades of toxicity.

### Response assessment

Computed tomography imaging was used to assess response as in Table 3. This was repeated 1 and 3 months after completion of chemotherapy. Further clinical assessment continued 3-monthly for the first year, 6-monthly for 5 years and annually thereafter. Symptoms relating to cardiac status were elicited on each occasion.

### Statistical considerations

This trial had a 2 × 2 factorial design and the primary end point for both randomisations was failure-free survival (FFS) defined as the time from the date of randomisation to the date of progression or death from disease, including non-responders, or death from treatment toxicity. Secondary end points for the comparison of CHOP with PMitCEBO and for the addition of G-CSF were response rates, toxicity, progression-free survival (PFS) and overall survival (OS). Progression-free survival was calculated from the date of randomisation to the date of progression or death from any cause whichever occurred first. Duration of OS was calculated from the date of randomisation to the date of death from any cause. At the time of the analysis, survivors were censored at the date they were last known to be alive. The log-rank test was applied to compare the Kaplan–Meier curves for FFS, PFS and OS (Kaplan and Meier, 1958). The standard χ^2^ test for frequency was used to compare response rate (CR) of treatment. Additional secondary end points for the G-CSF intervention were the number of in-patient days with and without sepsis.

The trial was powered to detect a 10% difference in the primary end point of FFS (assuming that the 3 year FFS with CHOP alone would be 30%), with a power of 90% and a significance level of 5% in a two-sided log-rank test for each of the two variables. Thus, the calculated sample size was 880 patients (440 in each arm). All analyses were on an intention to treat basis.

## RESULTS

Over 6 years, 784 eligible patients were entered into the trial. It was then closed prematurely following guidance from the UK National Institute for Clinical Excellence (NICE) that Rituximab should be given in combination with chemotherapy in all patients with diffuse large B-cell lymphoma. The median age of patients entered was 70 years (range 60–89). There were 411 (52%) male and 373 (48%) female patients. The proportion of patients with age adjusted international prognostic index risk (IPI) scores of 0, 1, 2, 3 was 16, 30, 37 and 17%, respectively. Over half the patients thus had poor prognostic disease.

Of these eligible patients, 195 patients were randomised into the CHOP arm, 202 into the PMitCEBO arm, 192 into the CHOP+G-CSF arm, and 195 into the PMitCEBO+G-CSF arm. The four cohorts were well balanced for all clinical characteristics (Table 4). Histological subtypes were well matched between the four cohorts. T-cell immunophenotype was seen in eleven cases in the CHOP arm, six cases in the PMitCEBO arm, seven cases in the CHOP+G-CSF arm and nine cases in the PMitCEBO+G-CSF arm. Analysis of the primary end point of FFS, revealed no significant interaction between the two randomisations (*P*=0.22) and the trial has therefore been analysed in accordance with the 2 × 2 factorial design, comparing the two CHOP cohorts with the two PMitCEBO cohorts and the two regimens with and without G-CSF. Data on the individual four treatment arms are only shown for reasons of exploratory or clinical information.

Of the CHOP recipients, 25% of patients received less than six cycles of chemotherapy predominantly due to early treatment failure. In total, 59% of patients received six cycles and 16% received more than this. In the PMitCEBO arm, 15% received less than eight cycles, 65% received 8–12 cycles, and 19% received 13–16 cycles. Of the patients randomised to receive G-CSF, it was given in 99% and of those randomised not to receive G-CSF, it was given in 8%; 17 patients in the CHOP arm and 12 patients in the PMitCEBO arm. In the majority of cases, G-CSF was given to maintain dose intensity due to haematological toxicity (fourteen patients) or as secondary prophylaxis (four patients). This was mainly initiated between courses 2 and 4. In the remaining cases, G-CSF was given due to a transcription error or the reason was not known. The use of radiation therapy was not stipulated in the protocol. Generally, involved field radiotherapy was given at the end of treatment to sites of initial bulk or residual disease. This was administered to 40 patients (10%) in the CHOP arm and 41 patients (10%) in the PMitCEBO arm.

### Response rates

The overall response rates for CHOP and PMitCEBO were almost identical at 84 and 83%, respectively (*P*=0.69), but the CR rate with CHOP (55%) was significantly higher than with PMitCEBO (47%, *P*=0.035).

The use of G-CSF had no impact on response rates with an overall response rate of 83% for the use of G-CSF and 84% for no G-CSF (*P*=0.84). The CR rates of 52% with G-CSF and 50% without G-CSF were similar (*P*=0.45).

### Survival

**Figure 1:**
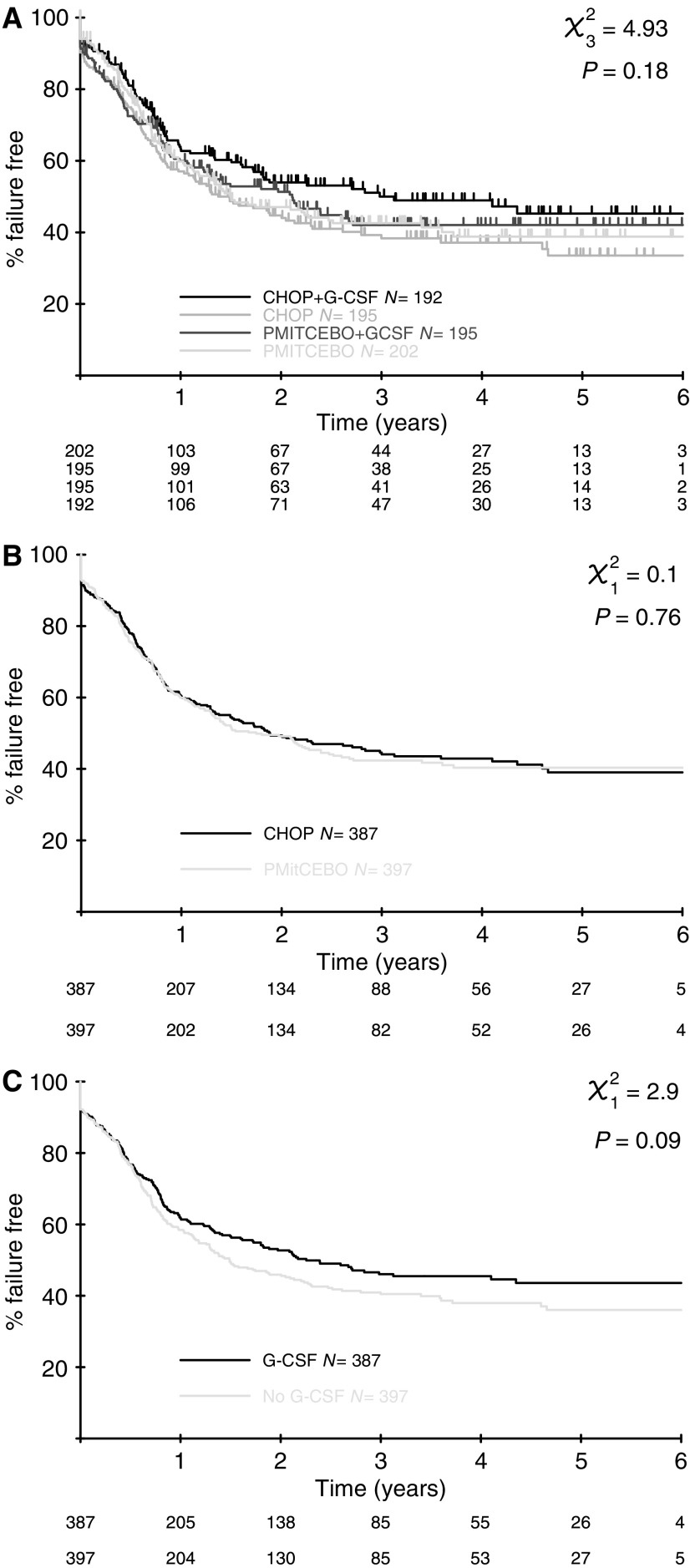
(**A**–**C**) Failure-free survival curves. (**A**) Failure-free survival all 4 arms; (**B**) failure-free survival CHOP *vs* PMitCEBO; (**C**) failure-free survival G-CSF *vs* no G-CSF.

**Figure 2:**
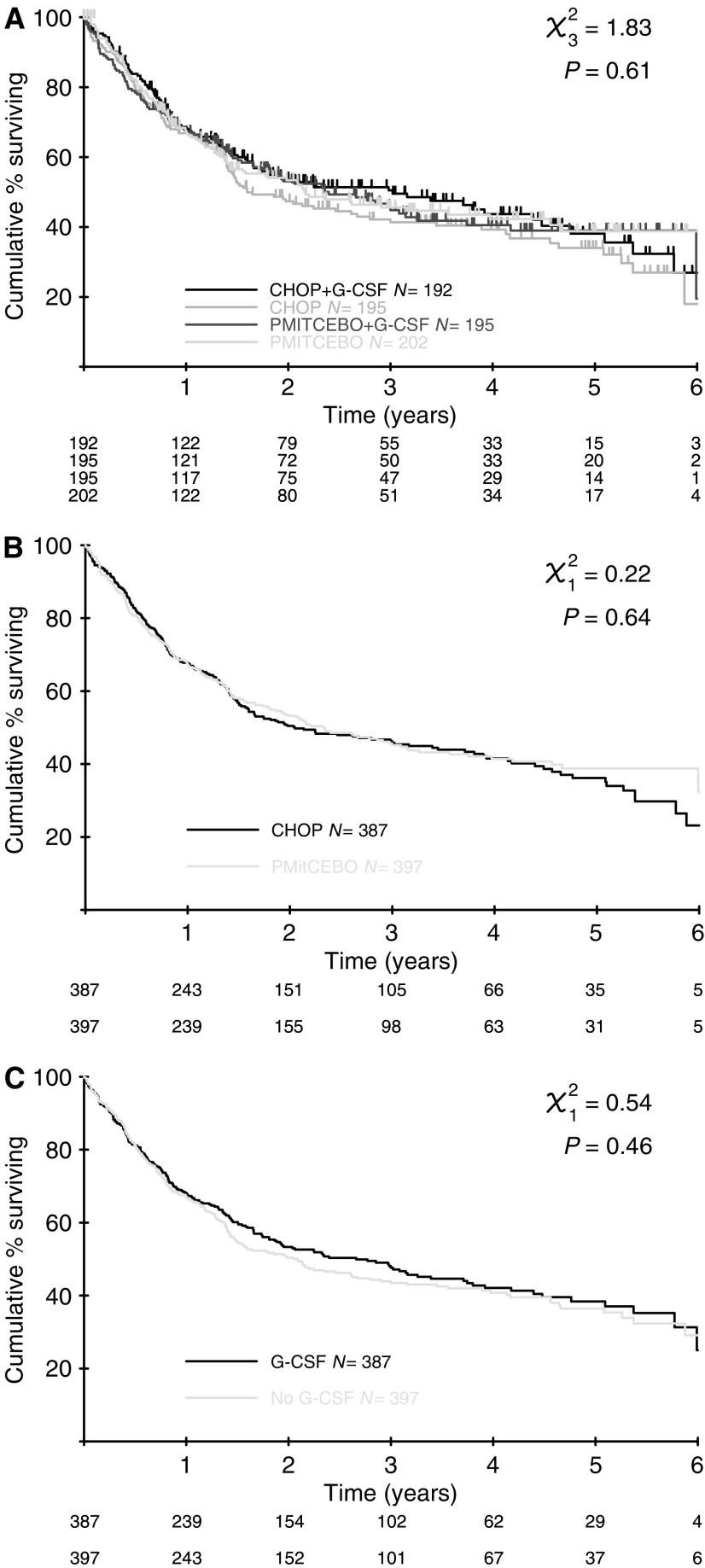
(**A**–**C**) Overall survival curves. (**A**) Overall survival all 4 arms; (**B**) overall survival CHOP *vs* PMitCEBO; (**C**) overall survival G-CSF *vs* no G-CSF.

At a median follow-up of 44 months, there was no significant difference in FFS between CHOP and PMitCEBO (*P*=0.76). At 3 years, the actuarial FFS was 44% in CHOP recipients and 42% in PMitCEBO recipients (Figure 1B). There was also no significant difference in PFS (*P*=0.77) or OS (*P*=0.57) (Figure 2B). The 3-year actuarial OS for patients receiving CHOP was 46% and for those receiving PMitCEBO was 45%. In total, 419 patients in this trial (53%) have died. In total, 318 were due to NHL, 158 in the CHOP arms and 160 in the PMitCEBO arms.

There was no significant difference in the FFS between those patients who received G-CSF and those who did not (*P*=0.23) (Figure 1C). Similarly, there was no significant difference in PFS (*P*=0.19) or OS (*P*=0.37) (Figure 2C). Of the deaths due to NHL, there were 147 among patients randomised to receive G-CSF and 171 among those who were not.

Subgroup analysis was performed for all patients according to IPI scores and age. Patients with IPI score 0 and 1 were compared with patients with an IPI score of 2 and 3 and patients <70 years were compared with patients ⩾70 years. The analysis was performed for CHOP *vs* PMitCEBO and G-CSF *vs* no G-CSF. Patients ⩾70 years had a reduced FFS and OS compared to patients of <70 years, but there were no significant differences between any randomised arms for either age cohort (Table 5). The poor prognosis patients also had a significantly reduced 3-year FFS and OS but no difference was apparent between randomisations (Table 5).

### Toxicity

The occurrence of grade 3 and 4 haematological toxicity was significantly more frequent in the PMitCEBO arm (63% of patients) compared with the CHOP arm (42%) (*P*<0.0001) (Table 6). This was mainly due to the increased incidence of leucopenia in the PMitCEBO arm (60%) compared to CHOP (39%) (*P*<0.0001) (Table 7). There was no increase in the maximum grade of infection toxicity reported with PMitCEBO (*P*=0.18) but these data must be treated with caution, as data on infection rates were incomplete. Hospitalisation rates and the number of days in hospital were comparable between CHOP and PMitCEBO (*P*=0.3) (Table 8). Gastrointestinal toxicity was significantly increased in the CHOP arm (*P*=0.008), especially nausea and vomiting (*P*=0.013). Alopecia was also more common in the CHOP arm, 69% of patients developing alopecia in the CHOP arm compared with 57% in the PMitCEBO arm (*P*=0.002). Grade 2–4 neuropathy was increased in the PMitCEBO arm (*P*=0.035). Acute and chronic cardiac toxicity were comparable between both chemotherapy regimens. No formal testing of cardiac function was performed prior to commencing treatment. It is acknowledged that further chronic cardiac toxicity may become apparent during prolonged follow-up. There have been 36 cardiac deaths, 17 in the CHOP arm, and 19 in the PMitCEBO arm (Table 9).

The addition of G-CSF significantly reduced the incidence of grade 3 and 4 haematological toxicity (*P*=0.024) due to a reduction in grade 3 and 4 leucopenia (*P*=0.001) (Tables 6 and 7). There was also a beneficial effect on the incidence of grade 3 and 4 anaemia associated with the use of G-CSF (*P*=0.004). The addition of G-CSF did not appear to impact on the maximum grade of infection toxicity (*P*=0.71), nor did it influence the hospitalisation rate or number of days in hospital (*P*=0.41) (Table 8). Almost all time spent in hospital was related to sepsis. An exploratory analysis has also been performed restricted to patients who received PMitCEBO, as this regimen was associated with more leucopenia. The proportion of patients treated with PMitCEBO without G-CSF who developed grade 3 or 4 leucopenia during treatment was 70% compared to 50% in those who received G-CSF (Table 7). There was no difference in infection toxicity or days in hospital (*P*=0.95) between the two groups.

## DISCUSSION

Special consideration of the management of elderly patients with aggressive NHL is necessary because of the increased risk of toxicity, morbidity and mortality from treatment and disease in this patient population. CHOP has been the most widely used regimen but treatment in the elderly is unsatisfactory, particularly when it is considered that many patients with co-morbidity, never receive such intensive combination chemotherapy regimens (Janssen-Heijnen *et al*, 2005). It is essential that regimens intended to reduce toxicity do not have a major negative impact on disease-response and this requires large trials, which have rarely been carried out in the elderly population. For this reason, the target recruitment for this trial was 880 patients and although the trial had to be terminated early, 89% of recruitment had occurred. Furthermore, the estimation of numbers required was based on an anticipated FFS at 3 years of 30% with the CHOP only regimen. In fact, the 3-year FFS in this arm was 44% and to see a 10% change from this baseline requires fewer patients, thus increasing the robustness of the results obtained.

The results of this trial are in accord with the poorer prognosis of older patients, but they also reaffirm that many elderly patients with histologically aggressive NHL can be cured of their disease. The 3-year overall survival for all patients of 46% compares favourably with other studies despite the high upper age-limit and the fact that over 50% of patients had poor prognostic disease as defined by the IPI (Osby *et al*, 2003; Pfreundschuh *et al*, 2004b; Feugier *et al*, 2005). Every effort should therefore be made to administer anthracycline-containing combination chemotherapy to elderly patients, if co-morbidity allows. Although outcome was worse in patients ⩾70 years, the overall survival at 3 years was over 35% justifying treatment with curative intent, although of course, increasing patient selection is inevitable with the older patients. The worst prognosis patient group, that is, ⩾70 years and IPI score 2–3, still achieved a 3 year overall survival of 28%.

This trial has demonstrated almost identical FFS and OS with CHOP and PMitCEBO. This equivalence of survival is despite the fact that the CR rate was less with PMitCEBO and presumably reflects that the earlier response assessment carried out with the shorter duration regimen underestimates the true response rate because of residual necrotic or fibrotic tissue (Surbone *et al*, 1988; Sweetenham *et al*, 1991). PMitCEBO is thus a valid alternative to CHOP contrasting with several other curtailed or modified regimens, which appear to be inferior (Meyer *et al*, 1995; Sonneveld *et al*, 1995; Bastion *et al*, 1997; Tirelli *et al*, 1998; Osby *et al*, 2003). A decision as to whether to use CHOP or PMitCEBO should therefore be based on the ease of administration and the comparative toxicities of the two regimens. PMitCEBO is given over a shorter treatment duration, although the number of hospital visits for injection of cytotoxic drugs is actually greater. PMitCEBO also induces less alopecia and less gastrointestinal disturbance. In addition, there is a reduced anthracycline dose with PMitCEBO. Assuming that 1 mg m^−2^ mitoxantrone equates to 5 mg m^−2^ doxorubicin (Posner *et al*, 1985), PMitCEBO contains about 30% less anthracycline than CHOP. This is potentially important in elderly patients in whom the incidence of cardiac toxicity may be higher than previously recognised and the threshold for damage may be within the range of the amount of doxorubicin given with CHOP (Swain *et al*, 2003).

The addition of prophylactic G-CSF to the two chemotherapy regimens, particularly PMitCEBO, resulted in a reduction in episodes of severe leucopenia in accord with previous studies (Zinzani *et al*, 1997; Doorduijn *et al*, 2003; Osby *et al*, 2003). This did not however impact on the rate of admission to hospital or the duration of hospitalisation, which reflect the incidence and severity of neutropenic sepsis. This concurs with the study of 389 patients reported by Doordujin *et al*, who found no impact of G-CSF on the incidence of infections or time in hospital in elderly patients receiving CHOP, although the studies by Osby and Zinzani *et al* found less infections and fewer days in hospital associated with G-CSF use. Most importantly, the use of G-CSF in this study did not improve the response rates, FFS or OS concordant with the studies referred to above. Taken together, this supports the assertion that primary prophylaxis with G-CSF is not indicated in elderly patients with NHL treated with regimens such as CHOP, although G-CSF is clearly required if CHOP is ‘time-escalated’ with reduction of the interval between cycles to 2 weeks (Wunderlich *et al*, 2003). It may also be warranted in selected individuals at particularly high risk for developing neutropenic sepsis (Balducci *et al*, 2001).

This trial was conducted before the advent of rituximab, and it is now widely accepted that rituximab should be given with chemotherapy in all cases of DLBC lymphoma (Pfreundschuh *et al*, 2004a; Feugier *et al*, 2005). This does not, however, detract from the finding in this study that PMitCEBO is an acceptable alternative to CHOP when the toxicity profile of PMitCEBO is preferable or more appropriate to the individual patient. The use of rituximab in addition to CHOP is associated with a marginal increase in neutropenia but not sufficient to affect the incidence of neutropenic fever or bacterial infections (Feugier *et al*, 2005). It is very unlikely, therefore, that the lack of clinical benefit found in this study with G-CSF use would be different with a rituximab containing regimen.

## Figures and Tables

**Table 1:** Treatment regimens for CHOP and PMitCEBO

**CHOP**	**PMitCEBO**
Cyclophosphamide 750 mg m^−2^ day 1	Cyclophosphamide 300 mg m^−2^ day 1
Doxorubicin 50 mg m^−2^ day 1	Mitoxantrone 7 mg m^−2^ day 1
Vincristine 1.4 mg m^−2^ day 1	Etoposide 150 mg m^−2^ day 1
Prednisolone 100 mg daily days 1–5	Prednisolone 50 mg daily weeks 1–4, 50 mg alternate days week 5 to treatment end
	Vincristine 1.4 mg m^−2^ day 8
	Bleomycin 10 mg m^−2^ day 8

**Table 2:** Dose modifications due to haematological toxicity, cycle 2 onwards

**Platelet count × 10^9^ l^−1^**	**Neutrophil count × 10^9^ l^−1^**	**Dose adjustment for CHOP**	**Dose adjustment for PMitCEBO**
100+	>1.5	100%	100%
75–99	1.0	75% cyclophosphamide, doxorubicin	100%
50–74	0.5–1.0	50% cyclophosphamide, doxorubicin	65% cyclophosphamide, mitoxantrone, etoposide
		100% vincristine	100% vincristine, bleomycin
<50	<0.5	Delay all drugs for 1 week	Delay all drugs for 1 week

**Table 3:** Criteria for response assessment

**Response**	**Definition**
CR	Resolution of all clinical and radiological abnormalities detected at presentation
PR	>50% resolution of all disease determined by product of two diameters
Non-progressive PR	No further reduction in previously responding disease
Progressive PR	Further reduction in previously responding disease
Nonresponse	<50% response subdivided into: (i) progressive disease if >50% increase in disease volume or development of new lesions; (ii)stable disease if disease status insufficient to meet criteria of partial response or progressive disease

**Table 4:** Patient characteristics

	**CHOP**	**PMitCEBO**	**CHOP+ GCSF**	**PMitCEBO+ GCSF**	**Total**
*Age (years)*					
Median	70	70	71	71	70
Range	60–86	60–89	60–87	60–85	60–89
					
*Sex (%)*					
Female	46	48	48	48	48
Male	54	52	52	52	52
					
*Stage (%)*					
I	9	8	11	10	10
II	29	30	24	23	27
III	29	25	29	34	29
IV	32	36	35	33	34
					
*B Symptoms (%)*					
A	49	43	50	42	46
B	51	57	50	58	54
					
*WHO PS (%)*					
0	32	31	34	30	32
1	41	43	37	38	40
2	17	18	20	26	20
3	8	6	6	4	6
4	2	2	2	3	2
					
*LDH (%)*					
Not raised	39	43	34	36	38
Raised	61	57	66	64	62
					
*IPI (%)*					
0	18	20	16	11	16
1	30	30	28	33	30
2	39	34	37	37	37
3	13	16	19	18	17

**Table 5:** 3 year failure-free and overall survival according to age and IPI score

	**CHOP**	**PMitCEBO**	**GCSF**	**No GCSF**
*3 year failure-free survival*				
Age (years)	%	%	%	%
<70	48	47	50	45
⩾70	40	38	42	36
				
IPI score				
0–1	56	57	56	55
2–3	36	24	34	26
				
*3-year overall survival*				
Age years	%	%	%	%
<70	53	52	54	51
⩾70	39	38	41	36
				
IPI score				
0–1	61	61	63	58
2–3	37	29	34	33

**Table 6:**
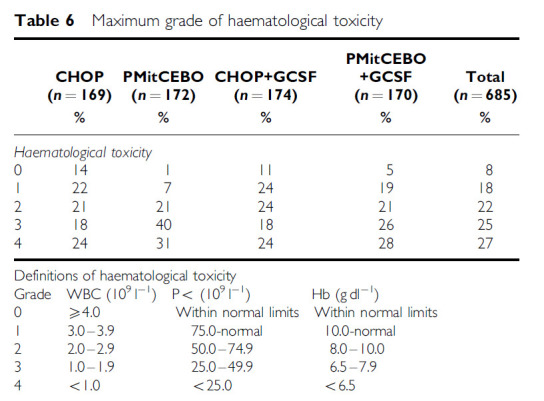
Maximum grade of haematological toxicity

**Table 7:**
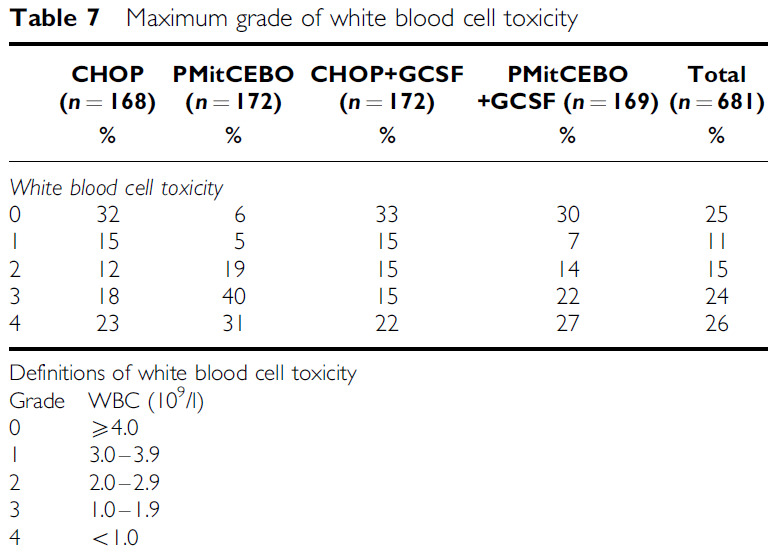
Maximum grade of white blood cell toxicity

**Table 8:** In-patient days

	**CHOP**	**PMitCEBO**	**CHOP+GCSF**	**PMitCEBO+GCSF**	**Total**
*In-patient days*	%	%	%	%	%
0	42	46	45	46	45
1–5	12	16	18	21	17
6–10	12	11	8	9	10
11–20	10	7	9	8	9
21–30	5	6	4	3	4
>30	9	6	8	6	7
					
Missing data	11	8	7	8	8

**Table 9:** Causes of death

	**CHOP**		**PMitCEBO**		**CHOP+GCSF**		**PMitCEBO+GCSF**		**Total**	
Cause of death	*N*	%	*N*	%	*N*	%	*N*	%	*N*	%
NHL	87	77	84	80	71	71	76	76	318	76
Other malignancy	7	6	1	1	2	2	2	2	12	3
										
*Death on treatment*										
Infection	4	4	5	5	2	2	1	1	12	3
Cardiac	5	4	2	2	2	2	7	7	16	4
Other	1	1	2	2	4	4	4	4	11	3
Unknown	0	0	0	0	0	0	0	0	0	0
										
*Death after treatment*										
Infection	2	2	2	2	4	4	2	2	10	2
Cardiac	3	3	7	7	7	7	3	3	20	5
Other	1	1	2	2	3	3	5	5	11	3
Unknown	3	3	0	0	4	4	0	0	7	2
Total^a^	113	100	105	100	99	100	100	100	417	100

a In total, 419 patients died. For two patients, who died from infection, it is not clear from the data whether they died on or after treatment (one in CHOP arm, one in CHOP+G-CSF arm).
